# Barriers and facilitators of community-based implementation of evidence-based interventions in the UK, for children and young people's mental health promotion, prevention and treatment: rapid scoping review

**DOI:** 10.1192/bjo.2023.531

**Published:** 2023-07-24

**Authors:** Abigail Thomson, Elinor Harris, Araminta Peters-Corbett, Keili Koppel, Cathy Creswell

**Affiliations:** Department of Psychiatry, University of Oxford, UK; Bodleian Health Care Libraries, University of Oxford, UK; Children and Young People's Mental Health Service, Norfolk and Suffolk NHS Foundation Trust, UK

**Keywords:** Evidence-based treatment, community-based services, children and young people, implementation, mental health

## Abstract

**Background:**

Community-based organisations continue to take on a greater role in supporting children and young people in the UK with their mental health. However, little evidence exists on the capacity and capability of these settings to effectively implement evidence-based interventions (EBIs).

**Aims:**

To identify barriers and facilitators of the implementation of EBIs within community settings in the UK, for children and young people's mental health promotion, prevention and treatment.

**Method:**

A PRISMA-guided, rapid scoping review was conducted, using predefined criteria and a relevant search strategy on eight databases: Ovid EMBASE, Ovid Medline, Ovid PsycINFO, Ovid Global Health: Scopus, Web of Science All Databases, EBSCO CINAHL and EBSCO ERIC. Study characteristics and data on barriers and facilitators were extracted, with results narratively synthesised.

**Results:**

Five out of 4899 studies met the inclusion criteria, addressing the barriers and facilitators of community-based implementation of EBIs for children and young people's mental health promotion, prevention and treatment. All of the studies that were identified focused on school settings, but we identified no studies that included data on barriers or facilitators of implementing EBIs in other community-based or voluntary sector settings.

**Conclusions:**

There is a lack of available evidence on the capacity and capability of community settings in the UK to effectively implement EBIs and adhere to evidence-based practice. However, existing findings within schools have highlighted key barriers and facilitators to implementation, such as the importance of meaningful involvement of stakeholders throughout the research process, and greater allocation of resources to support evidence-based decision-making in these settings.

Over the past decade, the UK has seen a significant increase in the prevalence of mental health problems in children and young people.^1^ One in six (16.0%) children in England aged 5–16 years have been identified as having a probable mental disorder – an increase from one in nine (10.8%) in 2017. Alongside this rising prevalence, there has been a subsequent increase in demand for clinical, voluntary and statutory services to support children and young people with their mental health. In an NHS Providers Survey carried out in May 2021, 100% of Mental Health Trust leaders said that the demand their systems were experiencing for services for children and young people had significantly (80%) or moderately (20%) increased compared with the previous 6 months.^2^ With the problem likely exacerbated by the COVID-19 pandemic and associated lockdowns, the need to increase provision for children and young people's mental health is now in sharp focus.

## The implementation of evidence-based interventions

A growing body of research is evolving aimed at supporting the promotion of mental health, prevention of emotional distress and development of treatment interventions to meet this need.^3^ Evidence-based interventions (EBIs) now exist for use across a wide range of clinical and non-clinical settings seeking to support children and young people. Those working within the voluntary and community sector (VCS) have been called upon by the UK Government in recent years to provide more visible and accessible support for children and young people.^4^ This includes voluntary and community groups, charities and third-sector organisations, all of which play a critical role for families and young people. Schools are similarly becoming recognised as community providers of mental health support, in what has been termed the ‘therapeutic turn’ in education.^5^ EBIs exist within these settings and have the potential to increase access to mental health guidance and support, particularly for those disadvantaged children and young people who otherwise would not receive help.^6^ Yet, despite a widespread call within the field to increase the role of community settings, such as schools and the VCS, in providing support for children and young people, little evidence exists on the capacity and capability of these settings to effectively implement EBIs and adhere to evidence-based practice.^7^

Previous research shows that very few studies to evaluate EBIs within community settings adequately report on implementation, adaptation and fidelity within their findings,^8^ despite reports that greater fidelity to intervention models is typically associated with better outcomes.^9^ In those studies that do report on implementation, several barriers and facilitators are emerging (i.e. lack of capacity within the workforce; lack of funding to meet high levels of demand; the leadership, level and quality of support available and the organisational structure), but their specific relevance for community services supporting children and young people has yet to be determined.^8^

Youth services in the UK are facing a specific set of challenges, including significant cuts to funding beyond that of adult services.^10^ It is essential that implementers within community settings in the UK – be it youth workers, teachers, volunteers, parents or even young people themselves – receive practical and relevant guidance and suggestions on how to implement EBIs, with this context in mind.

## Rationale

Effective implementation of EBIs at scale presents a challenge for those working to support children and young people, yet it is critical for the delivery of optimal and sustainable outcomes for families accessing services.^8^ The increasing prevalence of mental health problems in children and young people, and the rising demand on schools and the VCS in the UK to provide support, adds further urgency to better understand how evidence informs practice in these settings and address gaps between evidence and implementation. Therefore, this rapid scoping review of the literature sought to explore factors influencing the implementation of EBIs within UK-based and youth-focused community settings. Although existing research has examined implementation within the VCS globally, supporting individuals across ages,^7^ this review will adopt a narrower focus and seek to understand how evidence informs practice in UK-based, youth-focused community services. A scoping review was selected as it is well-equipped to map the available evidence in this field, and to identify key barriers and facilitators to implementation within the literature.^11^ The focus of this review is on those EBIs specifically targeted at children and young people aged 0–18 years, intending to promote, prevent or treat mental health problems within community settings. This review focuses on studies from the UK only, because of its specific organisational landscape, with the aim of better understanding the subsequent contextual determinants of barriers and facilitators facing researchers working to implement EBIs in the VCS and encourage future research in these settings. Given its focus, this review aimed to provide suggestions for ‘implementers’ that were most relevant to this unique context and considered the most appropriate evidence.

## Objectives

The main objectives of this review were (a) to identify barriers and facilitators of the implementation of EBIs in the UK, within community settings, for children and young people's mental health promotion, prevention and treatment; and (b) to draw on the identified literature to provide practical guidance and suggestions for ‘implementers’ within community settings in the UK, be it teachers, volunteers, parents or even young people themselves.

The focus of this rapid scoping review is captured in the following question: what are the barriers and facilitators of community-based implementation of EBIs in the UK, for children and young people's mental health promotion, prevention and treatment?

## Method

A rapid scoping review was conducted according to the procedure and requirements described in the Preferred Reporting Items for Systematic Reviews and Meta-Analyses Extension for Scoping Reviews (PRISMA-ScR). No protocol exists for this review.

### Search strategy

The search strategy was designed to be exhaustive in terms of identifying the current literature on studies investigating barriers to, and facilitators of, the delivery of EBIs outside of a clinical mental healthcare setting. A body of research addressing this topic was identified before the search through word of mouth, key reference lists and simple searches to check iterations of the strategy. The search was revised until it was sensitive enough to capture the preidentified studies. Eight databases were searched from 2011 to 30 July 2021: Ovid EMBASE, Ovid Medline, Ovid PsycINFO, Ovid Global Health: Scopus, Web of Science All Databases, EBSCO CINAHL and EBSCO ERIC.

A search strategy was constructed in collaboration with an information scientist (E.H.) to search for the relevant thesaurus headings in the Ovid databases, and free-text terms were applied to the title or abstract fields (or to all text fields in EBSCO ERIC) to search for synonyms for the following six concepts: children; evidence-based practice; mental health; community; implementation, barriers or facilitators; and the UK. The terms for evidence-based practice and barriers and facilitators were derived from Cahill et al,^12^ implementation terms were derived from Bach-Mortensen et al,^7^ mental health terms were derived from Uphoff et al^13^ and the Ovid EMBASE and Medline search strategies by Ayiku et al^14,15^ were used to retrieve research about the UK. These search strategies were then adapted for the other databases. Search results were limited to publications in the English language only. The full set of search strategies is available in Supplementary Appendix A available at https://doi.org/10.1192/bjo.2023.531.

All references were exported to EndNote for MacOS Ventura 13.4.1 X9 (Thomson Reuters, New York, USA; see https://endnote.com/product-details), and duplicates were removed following the method described by Falconer,^16^ before being imported into Rayyan.ai online platform for further deduplication and screening.

### Selection criteria

To be eligible for inclusion, studies had to be primary research studies from peer-reviewed journals, which investigated the barriers and facilitators of implementation of EBIs or programmes for the promotion, prevention and treatment of mental health in children and young people. Studies reporting on the implementation of EBIs and/or programmes targeted at participants aged 0–18 years and delivered outside of a clinical setting (by either a clinical or non-clinical professional) were considered.

In this review EBIs were defined as ‘interventions that have empirical support for improving mental health outcomes, which have been tested and validated according to the principles of evidence-based practice (e.g., having established feedback mechanisms between services and outcomes, the inclusion of stakeholders, and the best available evidence in decision-making processes)’. This definition has been validated in previous reviews of EBIs in the VCS.^7^ The population of study included young people, practitioners, commissioners and policymakers from within schools or community-based settings. This includes education practitioners, regional education authorities, third-sector organisations, social services, welfare services, local authorities, community leaders, police and crime commissioners. ‘Community-based’ was understood as any organisation that operates outside of the National Health Service, or other public or private healthcare providers (i.e. primary care practitioners would not be included).

Studies were included if the data was collected within the UK between 2011 and 2021. As previously mentioned, although global literature can provide important insights, in the case of this review, the objectives were to inform national guidance and provide suggestions for ‘implementers’. Therefore, focusing this search within the UK in the past 10 years would likely offer a better overview of the specific landscape, current challenges and considerations faced by schools and VCS within this country. For example, in the UK, there have been significant funding cuts to the VCS since 2010,^10^ and subsequent resourcing and capacity challenges. Overall, this review considers the evidence most relevant to this UK context.

To identify eligible papers, two raters independently reviewed titles and abstracts against the inclusion criteria (A.T. and A.P.-C. or K.K.). There was strong agreement (95–96%) between the screener pairs. A.T. retrieved relevant articles for full-text screening, and a random 10% of the included studies were also cross-checked at the full-text screening stage by A.P.-C. The full-text screening allowed for the exclusion of those papers investigating interventions or programs that are not evidence-based or do not provide data on barriers or facilitators to implementation. Disagreements (*n* = 1) were discussed, and a consensus was reached in line with the decision made by the primary screener.

### Data extraction

For those studies that aligned with the objectives and research question of the scoping review, data were extracted and charted by A.T. to record the following key information from each source: publication year and author; study aim (outcomes of interest, the target of intervention); methods (study design, data collection methods, data analysis); population (type of organisations, participant characteristics and sample size); the focus of EBIs implemented (universal, targeted); and results (barriers and facilitators in implementing EBIs).

### Data synthesis

Relevant barriers and facilitators were mapped and presented within tables. In line with the aims of a scoping review,^17^ a basic descriptive analysis and narrative overview is provided in relation to the research question. School-based studies are separated from studies based in other community-based organisations at this stage, because of the organisational differences that exist within school settings.

## Results

**Fig. 1:**
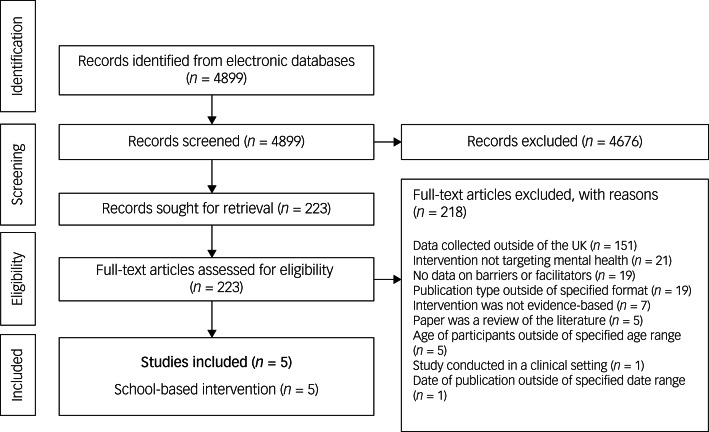
Review search and selection process.

The literature search resulted in 4899 citations (Fig. 1), of which 4676 were excluded at the title and abstract screening, the majority of which collected data outside of the UK. After screening the remaining 223 potentially relevant full-text papers, 150 were excluded as data was collected outside of the UK; 21 were excluded because the intervention targeted a concept unrelated to mental health promotion, prevention or treatment; 19 were excluded as they did not include data on barriers and facilitators of implementation and 19 were excluded because of incorrect publication type. Subsequently, only five papers were included.

### Study characteristics

A total of five studies met the inclusion criteria and were included in the review, all of which collected data on EBIs delivered in schools (Table 1). All studies were published between 2012 and 2020, included data on a universal intervention and used a qualitative analytic approach. The majority of studies interviewed staff delivering the interventions or working in the school, and only two collected data on the student experience.^18,19^ We did not identify any studies that included data on the barriers and facilitators of implementing EBIs in other community-based or voluntary sector settings.

**Table 1:** Study characteristics

(Study number) Author details	Year of publication	Study aim(s)	Method	Population	Focus of EBI implemented
(1) Edridge et al^18^	2019	To bridge the gap and examine the implementation of a mobile health intervention, ‘ReZone’ for reducing emotional and behavioural difficulties in young people in schools	Design: Study is part of a larger randomised controlled trial Data collection: Data was collected from students, on how often each intervention tool was used (adherence to intervention). Post-implementation consultations were carried out with teachers Data analysis: Framework analysis with determined topics was used to analyse consultations	Six participating schools (two primary and four alternative provision) 79 students (aged 10–15 years) 8 teachers	Universal treatment^a^
(2) Evans et al^22^	2015	To carry out a pragmatic formative process evaluation of the implementation of a social and emotional learning intervention	Design: Qualitative case analysis and evaluation Data collection: Semi-structured interviews Data analysis: Thematic analysis of interviews	4 participating schools from Wales 15 individuals were interviewed, including the intervention author and change agents (i.e. youth worker, teacher, school nurse)	Universal prevention
(3) Hudson et al^21^	2020	Identify the determinants of early implementation success of a mindfulness whole-school approach intervention, and discover if, how and why does the quality and extent of early implementation vary across schools	Design: Longitudinal qualitative study Data collection: Face-to-face or telephone interviews at two time points, 6 months apart Data analysis: Consolidated Framework for Implementation Research	5 participating schools from Cumbria 15 school staff interviewed	Universal promotion
(4) Wigelsworth et al^19^	2013	To analyse the impact of the SEAL programme, and assess the extent to which student outcomes vary as a function of implementation quality	Design: Quasi-experimental pre–post-test control group design/longitudinal case study Data collection: Strengths and Difficulties Questionnaire used to measure impact. Longitudinal case studies over 2 years to measure implementation – interviews with teachers and focus groups with students Data analysis: Summative judgements of implementation forming clusters – used as explanatory variables on which to model student outcomes	2442 pupils from 22 SEAL schools; 2001 pupils from 19 control schools A subsample of 9 of the 22 SEAL schools participated in additional data collection on implementation quality	Universal prevention
(5) Wilde et al^20^	2019	To develop an understanding of implementation of mindfulness in schools, from the perspective of key stakeholders (e.g. teachers, senior leadership)	Design: Multiple case study design Data collection: Semi-structured interviews Data analysis: Thematic analysis, using the PARiHS explanatory framework	7 secondary schools took part, across the UK 78 staff interviewed	Universal promotion

EBI, evidence-based intervention; PARiHS, Promoting Action on Research Implementation in Health Services framework; SEAL, Secondary Social and Emotional Aspects of Learning.

a. In this case, all students in participating schools were eligible to receive treatment.

### Barriers and facilitators to implementation

Each included study reported on barriers (Table 2) and facilitators (Table 3) to the implementation of an intervention.

**Table 2:** Barriers to implementation of evidence-based interventions delivered in schools

(Study number) Author details	Barriers to implementation identified
(1) Edridge et al^18^	Lack of resources to support use, including time and equipment Lack of engagement with teachers in the initial design and delivery of intervention Teachers’ busy schedules and competing priorities, such as examinations or academic targets High levels of time and support required from staff to monitor equipment usage Lack of engagement with parents/carers during the initial design and delivery of the intervention (i.e. parental consent forms not filled in, or parent requesting their child not take part) Lack of different stimuli and activities in the intervention app, which offered immediate rewards each time
(2) Evans et al^22^	Lack of acceptability of the intervention within the adoptive context, by teachers and staff delivering it Lack of fidelity to the intervention procedures by school staff, because of beliefs and preferences in delivery of certain components (e.g. support group) Poor level of intervention training delivered and teachers’ subsequent lack of technical knowledge of the intervention components Competing priorities and demands within schools Limited organisational capacity for sustained delivery of an intervention
(3) Hudson et al^21^	Low relative priority of the intervention compared with other school goals and targets Introducing multiple interventions simultaneously Knowledge and beliefs about the intervention and its effectiveness Fidelity to the intervention by school staff
(4) Wigelsworth et al^19^	Prioritisation of academic curriculum because of governmental pressure Organisational complexity of the secondary school setting presented barriers for consistency of delivery, reinforcement and communication
(5) Wilde et al^20^	Lack of fidelity to the intervention model by school staff as a result of high turnover and changes in leadership team Staff worries about safeguarding and lack of confidence in delivering the intervention High-cost and time-intensive training required to deliver the intervention Low relative priority of the intervention compared with other school goals and targets Fear of adverse effects of the intervention

**Table 3:** Facilitators to implementation of evidence-based interventions delivered in schools

(Study number) Author details	Facilitators to implementation identified
(1) Edridge et al^18^	App used was interactive in nature, and complimented other tools that students were already familiar with Availability of equipment (e.g. tablet to use app) Student actively seeking help with their emotions Implementing the intervention at the start of a new year was easier than trying to integrate it as a part of pre-existing school routines
(2) Evans et al^22^	Staff motivation to champion the intervention within their schools, as a result of interactive training workshops Intervention that can be quickly trialled, with outcomes easily observed Economic incentive to adopt the intervention Continued communication and clarification of intervention components and impact Broad network of support staff, implementing the intervention
(3) Hudson et al^21^	Engagement of leadership team with the intervention Effective networks of communication among staff Formally appointed intervention champions
(4) Wigelsworth et al^19^	Knowledge and beliefs about staff responsibility for mental health and well-being
(5) Wilde et al^20^	Formally appointed intervention champions Staff with specialist knowledge and training of the intervention Supportive senior leadership team The intervention fit well with the school culture and ethos, and could be easily embedded Positive perceptions of the intervention concept (e.g. mindfulness)

### Intervention-specific

Several intervention-specific barriers and facilitators to implementation were noted. Researchers highlighted the importance of integrating the research team in the delivery of the intervention or improving points of contact with the investigators, within the environment in which the intervention is being promoted and delivered.^20,21^ Researchers believed a lack of fidelity to the intervention model occurred as a result of poor points of contact with schools delivering it, and noted that many school staff opted to deliver only specific components of the intervention that they felt were most helpful or felt most confident in.^21^ Indeed, in all of those studies included, those advocating for an intervention and its fidelity were rarely the same people who implemented it daily.

It appeared that interventions were more effectively implemented if they could be easily embedded into current routines, practices or tools; for example, by implementing an intervention at the start of the school year, rather than partway through when routines have been established.^18^ Similarly, interventions using tools that students were already familiar with, and could access when needed, were much more successful in their implementation than interventions using novel technology that was restricted by school staff.^18^ Actively engaging students, parents, carers and teachers in the intervention was identified as especially important for encouraging sustained implementation and adherence to evidence-based practice. For example, in those EBIs that relied on parental consent, where researchers failed to engage parents and carers, implementation of the EBI within the school community was hindered.^18^ Similarly, in those interventions which drew upon existing knowledge and skills present in schools already, championing of the interventions within the school community and long-term implementation were supported.^19^ Other factors that affected the adherence to evidence-based practice included the complexity of the intervention, with staff commenting that more sophisticated and complex procedures had a negative effect on the acceptability of the approach and their confidence in delivery.^22^ More complex interventions similarly required more training, which many school staff did not have the capacity for.^18,22^ Other studies noted the value of not wholly relying on teachers to enable the implementation of intervention tools because of limited teacher capacity to supervise students’ use of the tool, but rather to include student self-directed intervention use.^18^

### Organisational

Several barriers and facilitators at a school level were also identified. In particular, limitations in school-wide capacity, readiness and willingness were highlighted as potential barriers to implementation across all of the studies. Although each study faced similar systematic challenges with capacity and resourcing, differences in implementation occurred as a result of each school's vision and ethos concerning mental health and well-being, which, in some cases, encouraged resource allocation toward supporting the implementation of the intervention.^19,20^ Further school-specific facilitators of implementation included the active support of (or lack of active obstruction from) the senior leadership within a school.^20^ The type of school within which the intervention was implemented was also highlighted.^18^ For example, the organisational complexity of a secondary school setting presented barriers to the consistency of delivery, reinforcement of intervention procedures, communication of intervention utility and, ultimately, adherence to the evidence base.^19^ Similarly, intervention delivery in alternative provisions presented extra challenges, as students required a greater number of support staff for delivery, which, for some interventions, was deemed to be unsustainable.^18^ Indeed, the availability of staff and resources within the school context was a challenge across all studies.

### Systemic

Barriers and facilitators also occurred as a result of education-specific systemic challenges. The most frequently noted challenge across all studies included a need to prioritise the academic curriculum and resources away from the intervention as a result of steep targets and governmental pressure. All schools struggled to combat this barrier when implementing interventions in the long term and adhering to evidence-based practice.

## Discussion

Five studies were identified that addressed the barriers and facilitators of community-based implementation of EBIs for children and young people's mental health promotion, prevention and treatment. All identified studies focused on school settings and, unfortunately, no studies were found that included data on the barriers or facilitators of implementing EBIs in wider community-based or voluntary sector settings.

### Barriers and facilitators

There were many similarities in the identified barriers and facilitators to implementation across the included studies. Perceived barriers and facilitators related to three domains: intervention-specific, organisational and systemic. With regard to intervention-specific barriers, the lack of active engagement and open communication with stakeholders was identified as a perceived barrier across all included studies. Engaging with the whole school community, including parents/carers, school staff and young people, was deemed to be paramount in ensuring the long-term implementation of an intervention within schools. By working closely with schools, understanding each school's ethos and the existing skills, knowledge and resources they have to offer, interventions are much more likely to be championed by stakeholders and within the school community. This could potentially aid evidence-based practice by encouraging resources and funding for mental health programmes, often overcoming many of the systemic barriers noted within schools.^19^ It is critical that researchers attempting to implement EBIs within a school setting actively engage and communicate with parents, carers and school staff, to ensure its acceptability within this context. Such a process might also aid fidelity to the intervention model if stakeholders and implementers feel able to communicate openly about the specific challenges they are facing, and the adaptations required to effectively implement the intervention.

Indeed, the organisational context of schools presented a barrier to implementation in itself. Unsurprisingly, all of the included studies identified challenges with school-wide capacity, readiness and willingness to implement EBIs. However, for some studies, the impact of such challenges was reduced as a result of the school's positive ethos and culture surrounding mental health and well-being.^19,20^ Although this was largely out of the researchers’ control, the recent ‘therapeutic turn’ in education^5^ may encourage more schools to allocate resources toward implementing universal EBIs for mental health and well-being. This may also improve fidelity to the intervention model in those instances where the required number of resources is high. It is also important to recognise the additional challenges that different types of schools face when implementing EBIs, including alternative provisions.^18^ Alternative provisions oversee some of the most vulnerable children and young people, many of whom seek support from both clinical and non-clinical settings.^23^ In these settings, students require a greater number of support staff for EBI delivery, which may be unsustainable in the long term with currently available resources.^18^ As such, researchers should be mindful of, and seek ways to, sustain the implementation of EBIs where a greater level of resource and support is required, to assist in effective implementation and evidence-based practice within settings such as alternative provisions.

Barriers and facilitators were also identified at a systemic level within the education system. Despite recent shifts and encouragement by the UK Government to offer more visible and accessible support for children and young people in the context of schools and the VCS,^4^ the reality of funding cuts and governmental pressure to meet steep academic targets encouraged resource allocation away from EBIs. This presented a barrier to implementation that all studies and schools struggled to combat. Notably, those initiatives that were encouraged by the UK Government (the Secondary Social and Emotional Aspects of Learning programme) faced similar challenges in prioritisation and implementation.^19^ To overcome this barrier it is key that interventions are developed with the involvement of the school community (i.e. teachers, parents, carers and young people) who understand and work with these systemic challenges on a daily basis. Being willing to work closely with stakeholders as an intervention is designed and trialled could create solutions to potential barriers before they arise.

### Implications

This review identified that the barriers and facilitators to the implementation of evidence-based practice for youth mental health in school settings are similar to those emerging within the wider literature, including resource challenges, level and quality of support available, and organisational structure.^8^ There were both within- and between-school variations for implementation quality. There was no difference in terms of the type of intervention being implemented (i.e. promotion, prevention, treatment). Notably, although all of those included studies commented on the barriers and facilitators of implementation within schools, none offered any pragmatic tools or guidance to optimise evidence-based practice in these settings. To support evidence-based practice in community-based services, future research should go beyond developing an understanding of the barriers and facilitators faced by implementers, and instead develop pragmatic implementation tools and frameworks for use in schools and the VCS.

Nevertheless, the findings of this review do highlight a number of challenges that UK implementers must be aware of when engaging with mental health interventions in their setting (e.g. lack of resources and capacity, poor level of intervention training). They further offer some potential solutions to overcome some of these barriers (e.g. appointing intervention champions, engaging leadership teams, economic incentives), all of which one can be mindful of in the absence of any pragmatic tools or frameworks at this stage. This will be especially pertinent as these settings are increasingly called upon to support the mental health of children and young people in the community.^4^ There is a clear need to develop research in this area and support new initiatives to implement evidence-based mental health practice within schools and community-based organisations. It is crucial to engage with stakeholders throughout these initiatives, and throughout the design, delivery, dissemination and implementation of an intervention.^24,25^

### Limitations of this review

It is important to highlight several limitations of this review. First, the lack of studies identified limited conclusions that could be drawn. There was a deliberate focus on studies from the UK only, given the likely contextual determinants of barriers and facilitators of implementation of EBIs. However, it is clear from these findings that there is not yet a sufficient evidence base within the UK, and research should turn toward the international evidence base in the meantime.

Similarly, for pragmatic reasons and in line with the rapid scoping review methodology, grey literature was not considered in this review. The authors recognise that reports on EBIs in the UK VCS are likely to exist beyond the peer-reviewed evidence base, and had these reports been included in this review, such additional perspectives may have changed the overall conclusions drawn from this study. The lack of reports from the VCS that were identified in the peer-reviewed literature in this study reflects a disconnect between academic research(ers) and the VCS – an important finding to consider. To bridge that gap, future research should make greater efforts to consider perspectives from the VCS that exist outside of the peer-reviewed evidence base, integrating findings across a range of disciplines to better support young people with their mental health.

Similarly, in future follow-ups, researchers should consider the nature of community-based settings and how they operate. Interventions are often in the earlier stages of development than those implemented in more clinical settings, and therefore adopting less stringent criteria on what is deemed to be ‘evidence-based’ may be useful in fully capturing the types of interventions being implemented in these settings. For example, there is a publication bias toward professionally led interventions, whereas many small and successful community health projects operate under the radar of formal evaluations.^26^

In addition, the dynamic nature of participatory methods being utilised in the VCS is not always reflected in academic literature, and often changes occur long after the evaluators have left.^27^ More evidence needs to be based on the lived experiences of those most affected by health inequalities, including through research controlled and led by patients/participants. In this review, only two of the included papers collected data on students’ experiences of the EBI being implemented.^19,20^ Young people have commonly been excluded and their voices have been absent from the research process in the past.^28^ Although this is changing, in cases where involvement with children and young people exists, it has sometimes been tokenistic and lacking in effectiveness.^28^ Future research should amplify the voices of young people and other stakeholders throughout the implementation process.

Finally, included studies were limited to shared characteristics, including the setting within which they took place (schools), the type of intervention (universal) and the method of data collection (qualitative interviews). No studies were identified from the wider VCS. As such, the review is limited in its ability to address barriers and facilitators to the implementation of EBIs, beyond school-based universal interventions.

In conclusion, this review has highlighted a lack of available evidence on the capacity and capability of community settings in the UK to effectively implement EBIs and adhere to evidence-based practice.^7^ Although there were a number of limitations, largely because of the rapid scoping review methodology, these findings (or lack thereof) represent a dearth of understanding or representation in the scientific literature of implementation and community-based approaches. They demonstrate a disconnect between academic researchers, practice and people.

More attention is needed to understand how EBIs are transported, contextualised and implemented by community providers, to ultimately broaden access to mental health support and guidance for children and young people. Ultimately, as the demand for clinical services grows, community settings must be given the tools to support children and young people. As such, research is needed to promote the development and dissemination of practical and sustainable implementation tools and guidance to support these organisations and the professionals working within them. Improving efficient and effective implementation of EBIs within community settings will require meaningful involvement of stakeholders throughout the research process, greater allocation of resources and funding for research and development to support evidence-based decision-making in schools and other community-based organisations, as they continue to take on a greater role in supporting children and young people with their mental health.

## Data Availability

Data availability is not applicable to this article as no new data were created or analysed in this study.

## Supplementary material

For supplementary material accompanying this paper visit http://doi.org/10.1192/bjo.2023.531. click here to view supplementary material
