# Barriers and facilitators of communitybased implementation of evidencebased interventions in the UK, for children and young people's mental health promotion, prevention and treatment: rapid scoping review – ERRATUM

**DOI:** 10.1192/bjo.2024.715

**Published:** 2024-05-03

**Authors:** Abigail Thomson, Elinor Harriss, Araminta Peters-Corbett, Keili Koppel, Cathy Creswell

**Keywords:** Evidence-based treatment, community-based services, children and young people, implementation, mental health, erratum

This article was originally published with Elinor Harriss’ name misspelled. This has now been corrected and this erratum published.

The publisher apologises for the error.
