# Supplementary material for: Barriers and facilitators of community-based implementation of evidence-based interventions in the UK, for children and young people's mental health promotion, prevention and treatment: rapid scoping review
Source: BJPsych Open. 2023 Jul 24;9(4):e132. doi: 10.1192/bjo.2023.531 (PMC10375901; doi:10.1192/bjo.2023.531)
Supplement: Supplementary file 1 [file S2056472423005318sup001.docx]

**Appendix A**

**Database: Embase 1974 to present**

Search Strategy:

--------------------------------------------------------------------------------

1 exp child/ or exp adolescent/ (3549358)

2 (infan* or newborn* or new-born* or perinat* or neonat* or baby* or babies or toddler* or minor* or boy* or girl* or kid or kids or child* or adolescen* or juvenil* or youth* or teen* or pubescen* or pediatric* or paediatric* or peadiatric* or school* or prematur* or preterm* or "pre term*" or prepub* or pre-pub* or preschool* or pre-school* or kindergarten* or nursery* or preadolescen* or pre-adolescen* or "young people" or "young person*" or "young adult*" or "young man" or "young men" or "young woman" or "young women").ti,ab. (3998443)

3 1 or 2 (5235124)

4 implementation science/ (2119)

5 *information dissemination/ (5165)

6 translational research/ (19167)

7 ((research or evidence) adj2 (action or practice)).ti,ab. (59321)

8 (knowledge adj2 (application or broke* or creation or diffus* or disseminat* or exchang* or implement* or management or mobili* or translat* or transfer* or uptak* or utili*)).ti,ab. (23315)

9 (evidence* adj2 (exchang* or translat* or transfer* or diffus* or disseminat* or exchang* or implement* or management or mobil* or uptak* or utili*)).ti,ab. (21425)

10 (research* adj2 (diffus* or disseminat* or exchang* or transfer* or translation* or application or implement* or mobil* or transfer* or uptak* or utili*)).ti,ab. (39894)

11 4 or 5 or 6 or 7 or 8 or 9 or 10 (150744)

12 mental health/ or community mental health/ or psychological well-being/ (178487)

13 mental disease/ (233085)

14 exp anxiety disorder/ (264026)

15 exp mood disorder/ (555436)

16 (mental* or well-being or wellbeing or psycho* or psychiatr* or resilience or behaviour* or behavior* or stress* or anxiety or anxious or panic* or phobi* or mood* or depress* or "adjustment disorder*" or trauma* or (emotional adj (adjustment* or disorder*))).ti,ab. (4493971)

17 12 or 13 or 14 or 15 or 16 (4692631)

18 exp school/ (367146)

19 community/ (83850)

20 exp policy/ (301605)

21 community mental health service/ (299)

22 community integration/ (1146)

23 social welfare/ (19404)

24 public health/ (195224)

25 (school* or "social services" or communit* or advocacy* or commission* or policy or policies or "local authorit*" or "public health" or justice or welfare or educat* or local or district or college or charity or "charitable organi*" or "classroom-based" or "whole-school" or "voluntary sector*" or "voluntary organi*" or "third sector" or third-sector or non-profit* or "not for profit" or "social enterprise*" or TSO* or CBO* or VCO* or VCS* or "civic sector*" or "civil sector*" or "social sector*" or CSO* or "non-government*" or NGO* or NPO* or "civil society" or CCG*).ti,ab. (3336796)

26 18 or 19 or 20 or 21 or 22 or 23 or 24 or 25 (3723526)

27 (barrier? or facilitator?).ti,ab. (426100)

28 exp program evaluation/ (28026)

29 (program* adj2 (evaluat* or acceptability or appropriateness or effectiveness or efficacy or feasibility or impact or sustainability)).ti,ab. (28351)

30 (implement* or adopt*).ti,ab. (1041417)

31 27 or 28 or 29 or 30 (1446153)

32 exp United Kingdom/ (432302)

33 (national health service* or nhs*).ti,ab,in,ad. (391941)

34 (english not ((published or publication* or translat* or written or language* or speak* or literature or citation*) adj5 english)).ti,ab. (48755)

35 (gb or "g.b." or britain* or (british* not "british columbia") or uk or "u.k." or united kingdom* or (england* not "new england") or northern ireland* or northern irish* or scotland* or scottish* or ((wales or "south wales") not "new south wales") or welsh*).ti,ab,jx,in,ad. (3355568)

36 (bath or "bath's" or ((birmingham not alabama*) or ("birmingham's" not alabama*) or bradford or "bradford's" or brighton or "brighton's" or bristol or "bristol's" or carlisle* or "carlisle's" or (cambridge not (massachusetts* or boston* or harvard*)) or ("cambridge's" not (massachusetts* or boston* or harvard*)) or (canterbury not zealand*) or ("canterbury's" not zealand*) or chelmsford or "chelmsford's" or chester or "chester's" or chichester or "chichester's" or coventry or "coventry's" or derby or "derby's" or (durham not (carolina* or nc)) or ("durham's" not (carolina* or nc)) or ely or "ely's" or exeter or "exeter's" or gloucester or "gloucester's" or hereford or "hereford's" or hull or "hull's" or lancaster or "lancaster's" or leeds* or leicester or "leicester's" or (lincoln not nebraska*) or ("lincoln's" not nebraska*) or (liverpool not (new south wales* or nsw)) or ("liverpool's" not (new south wales* or nsw)) or ((london not (ontario* or ont or toronto*)) or ("london's" not (ontario* or ont or toronto*)) or manchester or "manchester's" or (newcastle not (new south wales* or nsw)) or ("newcastle's" not (new south wales* or nsw)) or norwich or "norwich's" or nottingham or "nottingham's" or oxford or "oxford's" or peterborough or "peterborough's" or plymouth or "plymouth's" or portsmouth or "portsmouth's" or preston or "preston's" or ripon or "ripon's" or salford or "salford's" or salisbury or "salisbury's" or sheffield or "sheffield's" or southampton or "southampton's" or st albans or stoke or "stoke's" or sunderland or "sunderland's" or truro or "truro's" or wakefield or "wakefield's" or wells or westminster or "westminster's" or winchester or "winchester's" or wolverhampton or "wolverhampton's" or (worcester not (massachusetts* or boston* or harvard*)) or ("worcester's" not (massachusetts* or boston* or harvard*)) or (york not ("new york*" or ny or ontario* or ont or toronto*)) or ("york's" not ("new york*" or ny or ontario* or ont or toronto*))))).ti,ab,in,ad. (2600141)

37 (bangor or "bangor's" or cardiff or "cardiff's" or newport or "newport's" or st asaph or "st asaph's" or st davids or swansea or "swansea's").ti,ab,in,ad. (106512)

38 (aberdeen or "aberdeen's" or dundee or "dundee's" or edinburgh or "edinburgh's" or glasgow or "glasgow's" or inverness or (perth not australia*) or ("perth's" not australia*) or stirling or "stirling's").ti,ab,in,ad. (357883)

39 (armagh or "armagh's" or belfast or "belfast's" or lisburn or "lisburn's" or londonderry or "londonderry's" or derry or "derry's" or newry or "newry's").ti,ab,in,ad. (48841)

40 32 or 33 or 34 or 35 or 36 or 37 or 38 or 39 (4093441)

41 (exp "arctic and antarctic"/ or exp oceanic regions/ or exp western hemisphere/ or exp africa/ or exp asia/ or exp "australia and new zealand"/) not (exp united kingdom/ or europe/) (3323445)

42 40 not 41 (3853954)

43 3 and 11 and 17 and 26 and 31 and 42 (309)

44 43 (309)

45 limit 44 to (english language and yr="2011 -Current") (261)

**Database: Medline (Ovid MEDLINE® Epub Ahead of Print, In-Process & Other Non-Indexed Citations, Ovid MEDLINE® Daily and Ovid MEDLINE®) 1946 to present**

Search Strategy:

--------------------------------------------------------------------------------

1 adolescent/ or exp child/ or exp infant/ (3708579)

2 (infan* or newborn* or new-born* or perinat* or neonat* or baby* or babies or toddler* or minor* or boy* or girl* or kid or kids or child* or adolescen* or juvenil* or youth* or teen* or pubescen* or pediatric* or paediatric* or peadiatric* or school* or prematur* or preterm* or "pre term*" or prepub* or pre-pub* or preschool* or pre-school* or kindergarten* or nursery* or preadolescen* or pre-adolescen* or "young people" or "young person*" or "young adult*" or "young man" or "young men" or "young woman" or "young women").ti,ab. (3230542)

3 1 or 2 (5048878)

4 implementation science/ (784)

5 *Information Dissemination/ (9814)

6 Translational Medical Research/ (11874)

7 ((research or evidence) adj2 (action or practice)).ti,ab. (45783)

8 (knowledge adj2 (application or broke* or creation or diffus* or disseminat* or exchang* or implement* or management or mobili* or translat* or transfer* or uptak* or utili*)).ti,ab. (17582)

9 (evidence* adj2 (exchang* or translat* or transfer* or diffus* or disseminat* or exchang* or implement* or management or mobil* or uptak* or utili*)).ti,ab. (16545)

10 (research* adj2 (diffus* or disseminat* or exchang* or transfer* or translation* or application or implement* or mobil* or transfer* or uptak* or utili*)).ti,ab. (30478)

11 4 or 5 or 6 or 7 or 8 or 9 or 10 (119647)

12 Mental Health/ (45533)

13 Mental Disorders/ (168496)

14 anxiety disorders/ or anxiety, separation/ or panic disorder/ or phobic disorders/ or phobia, social/ (52546)

15 mood disorders/ or depressive disorder/ or depressive disorder, major/ or depressive disorder, treatment-resistant/ (119795)

16 "trauma and stressor related disorders"/ or stress disorders, traumatic/ or psychological trauma/ or stress disorders, post-traumatic/ or stress disorders, traumatic, acute/ (37518)

17 (mental* or well-being or wellbeing or psycho* or psychiatr* or resilience or behaviour* or behavior* or stress* or anxiety or anxious or panic* or phobi* or mood* or depress* or "adjustment disorder*" or trauma* or (emotional adj (adjustment* or disorder*))).ti,ab. (3675255)

18 12 or 13 or 14 or 15 or 16 or 17 (3732765)

19 schools/ or schools, nursery/ (43918)

20 Residence Characteristics/ (35795)

21 policy/ or exp social control policies/ (163115)

22 Community Mental Health Services/ (18805)

23 Community Integration/ (395)

24 exp Social Welfare/ (59082)

25 exp Public Health/ (8350078)

26 (school* or "social services" or communit* or advocacy* or commission* or policy or policies or "local authorit*" or "public health" or justice or welfare or educat* or local or district or college or charity or "charitable organi*" or "classroom-based" or "whole-school" or "voluntary sector*" or "voluntary organi*" or "third sector" or third-sector or non-profit* or "not for profit" or "social enterprise*" or TSO* or CBO* or VCO* or VCS* or "civic sector*" or "civil sector*" or "social sector*" or CSO* or "non-government*" or NGO* or NPO* or "civil society" or CCG*).ti,ab. (2617895)

27 19 or 20 or 21 or 22 or 23 or 24 or 25 or 26 (9839556)

28 (barrier? or facilitator?).ti,ab. (342184)

29 Program Evaluation/ (65383)

30 (program* adj2 (evaluat* or acceptability or appropriateness or effectiveness or efficacy or feasibility or impact or sustainability)).ti,ab. (21004)

31 (implement* or adopt*).ti,ab. (808036)

32 28 or 29 or 30 or 31 (1165921)

33 exp United Kingdom/ (377124)

34 (national health service* or nhs*).ti,ab,in. (224289)

35 (english not ((published or publication* or translat* or written or language* or speak* or literature or citation*) adj5 english)).ti,ab. (41231)

36 (gb or "g.b." or britain* or (british* not "british columbia") or uk or "u.k." or "united kingdom*" or (england* not "new england") or "northern ireland*" or "northern irish*" or scotland* or scottish* or ((wales or "south wales") not "new south wales") or welsh*).ti,ab,jw,in. (2205371)

37 (bath or "bath's" or ((birmingham not alabama*) or ("birmingham's" not alabama*) or bradford or "bradford's" or brighton or "brighton's" or bristol or "bristol's" or carlisle* or "carlisle's" or (cambridge not (massachusetts* or boston* or harvard*)) or ("cambridge's" not (massachusetts* or boston* or harvard*)) or (canterbury not zealand*) or ("canterbury's" not zealand*) or chelmsford or "chelmsford's" or chester or "chester's" or chichester or "chichester's" or coventry or "coventry's" or derby or "derby's" or (durham not (carolina* or nc)) or ("durham's" not (carolina* or nc)) or ely or "ely's" or exeter or "exeter's" or gloucester or "gloucester's" or hereford or "hereford's" or hull or "hull's" or lancaster or "lancaster's" or leeds* or leicester or "leicester's" or (lincoln not nebraska*) or ("lincoln's" not nebraska*) or (liverpool not (new south wales* or nsw)) or ("liverpool's" not (new south wales* or nsw)) or ((london not (ontario* or ont or toronto*)) or ("london's" not (ontario* or ont or toronto*)) or manchester or "manchester's" or (newcastle not (new south wales* or nsw)) or ("newcastle's" not (new south wales* or nsw)) or norwich or "norwich's" or nottingham or "nottingham's" or oxford or "oxford's" or peterborough or "peterborough's" or plymouth or "plymouth's" or portsmouth or "portsmouth's" or preston or "preston's" or ripon or "ripon's" or salford or "salford's" or salisbury or "salisbury's" or sheffield or "sheffield's" or southampton or "southampton's" or st albans or stoke or "stoke's" or sunderland or "sunderland's" or truro or "truro's" or wakefield or "wakefield's" or wells or westminster or "westminster's" or winchester or "winchester's" or wolverhampton or "wolverhampton's" or (worcester not (massachusetts* or boston* or harvard*)) or ("worcester's" not (massachusetts* or boston* or harvard*)) or (york not ("new york*" or ny or ontario* or ont or toronto*)) or ("york's" not ("new york*" or ny or ontario* or ont or toronto*))))).ti,ab,in. (1530036)

38 (bangor or "bangor's" or cardiff or "cardiff's" or newport or "newport's" or st asaph or "st asaph's" or st davids or swansea or "swansea's").ti,ab,in. (60817)

39 (aberdeen or "aberdeen's" or dundee or "dundee's" or edinburgh or "edinburgh's" or glasgow or "glasgow's" or inverness or (perth not australia*) or ("perth's" not australia*) or stirling or "stirling's").ti,ab,in. (226122)

40 (armagh or "armagh's" or belfast or "belfast's" or lisburn or "lisburn's" or londonderry or "londonderry's" or derry or "derry's" or newry or "newry's").ti,ab,in. (28865)

41 33 or 34 or 35 or 36 or 37 or 38 or 39 or 40 (2771135)

42 (exp africa/ or exp americas/ or exp antarctic regions/ or exp arctic regions/ or exp asia/ or exp australia/ or exp oceania/) not (exp united kingdom/ or europe/) (3054492)

43 41 not 42 (2633561)

44 3 and 11 and 18 and 27 and 32 and 43 (294)

45 44 (294)

46 limit 45 to (english language and yr="2011 -Current") (234)

**Database: PsycINFO 1806 to present**

Search Strategy:

--------------------------------------------------------------------------------

1 (infan* or newborn* or new-born* or perinat* or neonat* or baby* or babies or toddler* or minor* or boy* or girl* or kid or kids or child* or adolescen* or juvenil* or youth* or teen* or pubescen* or pediatric* or paediatric* or peadiatric* or school* or prematur* or preterm* or "pre term*" or prepub* or pre-pub* or preschool* or pre-school* or kindergarten* or nursery* or preadolescen* or pre-adolescen* or "young people" or "young person*" or "young adult*" or "young man" or "young men" or "young woman" or "young women").ti,ab. (1347420)

2 evidence based practice/ (18985)

3 information dissemination/ (1890)

4 ((research or evidence) adj2 (action or practice)).ti,ab. (50141)

5 (knowledge adj2 (application or broke* or creation or diffus* or disseminat* or exchang* or implement* or management or mobili* or translat* or transfer* or uptak* or utili*)).ti,ab. (13486)

6 (evidence* adj2 (exchang* or translat* or transfer* or diffus* or disseminat* or exchang* or implement* or management or mobil* or uptak* or utili*)).ti,ab. (6156)

7 (research* adj2 (diffus* or disseminat* or exchang* or transfer* or translation* or application or implement* or mobil* or transfer* or uptak* or utili*)).ti,ab. (15447)

8 2 or 3 or 4 or 5 or 6 or 7 (93589)

9 exp mental health/ (73392)

10 exp mental disorders/ (898657)

11 exp trauma/ (84984)

12 (mental* or well-being or wellbeing or psycho* or psychiatr* or resilience or behaviour* or behavior* or stress* or anxiety or anxious or panic* or phobi* or mood* or depress* or "adjustment disorder*" or trauma* or (emotional adj (adjustment* or disorder*))).ti,ab. (2450472)

13 9 or 10 or 11 or 12 (2722415)

14 exp schools/ (72779)

15 communities/ (32384)

16 health care policy/ (11085)

17 exp community mental health services/ (7888)

18 nonprofit organizations/ (2710)

19 exp public health/ (36452)

20 (school* or "social services" or communit* or advocacy* or commission* or policy or policies or "local authorit*" or "public health" or justice or welfare or educat* or local or district or college or charity or "charitable organi*" or "classroom-based" or "whole-school" or "voluntary sector*" or "voluntary organi*" or "third sector" or third-sector or non-profit* or "not for profit" or "social enterprise*" or TSO* or CBO* or VCO* or VCS* or "civic sector*" or "civil sector*" or "social sector*" or CSO* or "non-government*" or NGO* or NPO* or "civil society" or CCG*).ti,ab. (1320277)

21 14 or 15 or 16 or 17 or 18 or 19 or 20 (1335484)

22 (barrier? or facilitator?).ti,ab. (88201)

23 exp program evaluation/ (21161)

24 (program* adj2 (evaluat* or acceptability or appropriateness or effectiveness or efficacy or feasibility or impact or sustainability)).ti,ab. (16318)

25 (implement* or adopt*).ti,ab. (279273)

26 22 or 23 or 24 or 25 (374352)

27 (national health service* or nhs*).ti,ab. (7027)

28 (english not ((published or publication* or translat* or written or language* or speak* or literature or citation*) adj5 english)).ti,ab. (97605)

29 (gb or "g.b." or britain* or (british* not "british columbia") or uk or "u.k." or united kingdom* or (england* not "new england") or northern ireland* or northern irish* or scotland* or scottish* or ((wales or "south wales") not "new south wales") or welsh*).ti,ab. (94265)

30 (bath or "bath's" or ((birmingham not alabama*) or ("birmingham's" not alabama*) or bradford or "bradford's" or brighton or "brighton's" or bristol or "bristol's" or carlisle* or "carlisle's" or (cambridge not (massachusetts* or boston* or harvard*)) or ("cambridge's" not (massachusetts* or boston* or harvard*)) or (canterbury not zealand*) or ("canterbury's" not zealand*) or chelmsford or "chelmsford's" or chester or "chester's" or chichester or "chichester's" or coventry or "coventry's" or derby or "derby's" or (durham not (carolina* or nc)) or ("durham's" not (carolina* or nc)) or ely or "ely's" or exeter or "exeter's" or gloucester or "gloucester's" or hereford or "hereford's" or hull or "hull's" or lancaster or "lancaster's" or leeds* or leicester or "leicester's" or (lincoln not nebraska*) or ("lincoln's" not nebraska*) or (liverpool not (new south wales* or nsw)) or ("liverpool's" not (new south wales* or nsw)) or ((london not (ontario* or ont or toronto*)) or ("london's" not (ontario* or ont or toronto*)) or manchester or "manchester's" or (newcastle not (new south wales* or nsw)) or ("newcastle's" not (new south wales* or nsw)) or norwich or "norwich's" or nottingham or "nottingham's" or oxford or "oxford's" or peterborough or "peterborough's" or plymouth or "plymouth's" or portsmouth or "portsmouth's" or preston or "preston's" or ripon or "ripon's" or salford or "salford's" or salisbury or "salisbury's" or sheffield or "sheffield's" or southampton or "southampton's" or st albans or stoke or "stoke's" or sunderland or "sunderland's" or truro or "truro's" or wakefield or "wakefield's" or wells or westminster or "westminster's" or winchester or "winchester's" or wolverhampton or "wolverhampton's" or (worcester not (massachusetts* or boston* or harvard*)) or ("worcester's" not (massachusetts* or boston* or harvard*)) or (york not ("new york*" or ny or ontario* or ont or toronto*)) or ("york's" not ("new york*" or ny or ontario* or ont or toronto*))))).ti,ab. (34013)

31 (bangor or "bangor's" or cardiff or "cardiff's" or newport or "newport's" or st asaph or "st asaph's" or st davids or swansea or "swansea's").ti,ab. (617)

32 (aberdeen or "aberdeen's" or dundee or "dundee's" or edinburgh or "edinburgh's" or glasgow or "glasgow's" or inverness or (perth not australia*) or ("perth's" not australia*) or stirling or "stirling's").ti,ab. (6896)

33 (armagh or "armagh's" or belfast or "belfast's" or lisburn or "lisburn's" or londonderry or "londonderry's" or derry or "derry's" or newry or "newry's").ti,ab. (479)

34 27 or 28 or 29 or 30 or 31 or 32 or 33 (222936)

35 1 and 8 and 13 and 21 and 26 and 34 (127)

36 35 (127)

37 limit 36 to (english language and yr="2011 -Current") (84)

**Database: Global Health <1973 to 2021 Week 30>**

Search Strategy:

--------------------------------------------------------------------------------

1 exp infants/ (122423)

2 exp children/ (324410)

3 exp adolescents/ (69294)

4 (infan* or newborn* or new-born* or perinat* or neonat* or baby* or babies or toddler* or minor* or boy* or girl* or kid or kids or child* or adolescen* or juvenil* or youth* or teen* or pubescen* or pediatric* or paediatric* or peadiatric* or school* or prematur* or preterm* or "pre term*" or prepub* or pre-pub* or preschool* or pre-school* or kindergarten* or nursery* or preadolescen* or pre-adolescen* or "young people" or "young person*" or "young adult*" or "young man" or "young men" or "young woman" or "young women").ti,ab. (627119)

5 1 or 2 or 3 or 4 (639892)

6 "diffusion of information"/ (2244)

7 ((research or evidence) adj2 (action or practice)).ti,ab. (6425)

8 (knowledge adj2 (application or broke* or creation or diffus* or disseminat* or exchang* or implement* or management or mobili* or translat* or transfer* or uptak* or utili*)).ti,ab. (3660)

9 (evidence* adj2 (exchang* or translat* or transfer* or diffus* or disseminat* or exchang* or implement* or management or mobil* or uptak* or utili*)).ti,ab. (2955)

10 (research* adj2 (diffus* or disseminat* or exchang* or transfer* or translation* or application or implement* or mobil* or transfer* or uptak* or utili*)).ti,ab. (4980)

11 6 or 7 or 8 or 9 or 10 (18729)

12 mental health/ (26846)

13 exp mental disorders/ (80382)

14 (mental* or well-being or wellbeing or psycho* or psychiatr* or resilience or behaviour* or behavior* or stress* or anxiety or anxious or panic* or phobi* or mood* or depress* or "adjustment disorder*" or trauma* or (emotional adj (adjustment* or disorder*))).ti,ab. (438301)

15 12 or 13 or 14 (457535)

16 exp schools/ (17724)

17 communities/ (15336)

18 exp policy/ (50967)

19 community health services/ (4387)

20 exp social welfare/ (2177)

21 public health/ (141153)

22 (school* or "social services" or communit* or advocacy* or commission* or policy or policies or "local authorit*" or "public health" or justice or welfare or educat* or local or district or college or charity or "charitable organi*" or "classroom-based" or "whole-school" or "voluntary sector*" or "voluntary organi*" or "third sector" or third-sector or non-profit* or "not for profit" or "social enterprise*" or TSO* or CBO* or VCO* or VCS* or "civic sector*" or "civil sector*" or "social sector*" or CSO* or "non-government*" or NGO* or NPO* or "civil society" or CCG*).ti,ab. (670362)

23 16 or 17 or 18 or 19 or 20 or 21 or 22 (722192)

24 (barrier? or facilitator?).ti,ab. (56474)

25 program evaluation/ (659)

26 (program* adj2 (evaluat* or acceptability or appropriateness or effectiveness or efficacy or feasibility or impact or sustainability)).ti,ab. (5601)

27 (implement* or adopt*).ti,ab. (169093)

28 24 or 25 or 26 or 27 (218885)

29 exp uk/ (79080)

30 (national health service* or nhs*).ti,ab. (5401)

31 (english not ((published or publication* or translat* or written or language* or speak* or literature or citation*) adj5 english)).ti,ab. (8835)

32 (gb or "g.b." or britain* or (british* not "british columbia") or uk or "u.k." or united kingdom* or (england* not "new england") or northern ireland* or northern irish* or scotland* or scottish* or ((wales or "south wales") not "new south wales") or welsh*).ti,ab. (70860)

33 (bath or "bath's" or ((birmingham not alabama*) or ("birmingham's" not alabama*) or bradford or "bradford's" or brighton or "brighton's" or bristol or "bristol's" or carlisle* or "carlisle's" or (cambridge not (massachusetts* or boston* or harvard*)) or ("cambridge's" not (massachusetts* or boston* or harvard*)) or (canterbury not zealand*) or ("canterbury's" not zealand*) or chelmsford or "chelmsford's" or chester or "chester's" or chichester or "chichester's" or coventry or "coventry's" or derby or "derby's" or (durham not (carolina* or nc)) or ("durham's" not (carolina* or nc)) or ely or "ely's" or exeter or "exeter's" or gloucester or "gloucester's" or hereford or "hereford's" or hull or "hull's" or lancaster or "lancaster's" or leeds* or leicester or "leicester's" or (lincoln not nebraska*) or ("lincoln's" not nebraska*) or (liverpool not (new south wales* or nsw)) or ("liverpool's" not (new south wales* or nsw)) or ((london not (ontario* or ont or toronto*)) or ("london's" not (ontario* or ont or toronto*)) or manchester or "manchester's" or (newcastle not (new south wales* or nsw)) or ("newcastle's" not (new south wales* or nsw)) or norwich or "norwich's" or nottingham or "nottingham's" or oxford or "oxford's" or peterborough or "peterborough's" or plymouth or "plymouth's" or portsmouth or "portsmouth's" or preston or "preston's" or ripon or "ripon's" or salford or "salford's" or salisbury or "salisbury's" or sheffield or "sheffield's" or southampton or "southampton's" or st albans or stoke or "stoke's" or sunderland or "sunderland's" or truro or "truro's" or wakefield or "wakefield's" or wells or westminster or "westminster's" or winchester or "winchester's" or wolverhampton or "wolverhampton's" or (worcester not (massachusetts* or boston* or harvard*)) or ("worcester's" not (massachusetts* or boston* or harvard*)) or (york not ("new york*" or ny or ontario* or ont or toronto*)) or ("york's" not ("new york*" or ny or ontario* or ont or toronto*))))).ti,ab. (32896)

34 (bangor or "bangor's" or cardiff or "cardiff's" or newport or "newport's" or st asaph or "st asaph's" or st davids or swansea or "swansea's").ti,ab. (1009)

35 (aberdeen or "aberdeen's" or dundee or "dundee's" or edinburgh or "edinburgh's" or glasgow or "glasgow's" or inverness or (perth not australia*) or ("perth's" not australia*) or stirling or "stirling's").ti,ab. (4759)

36 (armagh or "armagh's" or belfast or "belfast's" or lisburn or "lisburn's" or londonderry or "londonderry's" or derry or "derry's" or newry or "newry's").ti,ab. (241)

37 29 or 30 or 31 or 32 or 33 or 34 or 35 or 36 (128227)

38 5 and 11 and 15 and 23 and 28 and 37 (40)

39 38 (40)

40 limit 39 to (english language and yr="2011 -Current") (28)

**Scopus**

( TITLE-ABS-KEY ( ( infan* OR newborn* OR new-born* OR perinat* OR neonat* OR baby* OR babies OR toddler* OR minor* OR boy* OR girl* OR kid OR kids OR child* OR adolescen* OR juvenil* OR youth* OR teen* OR pubescen* OR pediatric* OR paediatric* OR peadiatric* OR school* OR prematur* OR preterm* OR "pre term*" OR prepub* OR pre-pub* OR preschool* OR pre-school* OR kindergarten* OR nursery* OR preadolescen* OR pre-adolescen* OR "young people" OR "young person*" OR "young adult*" OR "young man" OR "young men" OR "young woman" OR "young women" ) ) ) AND ( ( TITLE-ABS-KEY ( ( ( research OR evidence ) W/2 ( action OR practice ) ) ) OR TITLE-ABS-KEY ( ( knowledge W/2 ( application OR broke* OR creation OR diffus* OR disseminat* OR exchang* OR implement* OR management OR mobili* OR translat* OR transfer* OR uptak* OR utili* ) ) ) OR TITLE-ABS-KEY ( ( evidence* W/2 ( exchang* OR translat* OR transfer* OR diffus* OR disseminat* OR exchang* OR implement* OR management OR mobil* OR uptak* OR utili* ) ) ) OR TITLE-ABS-KEY ( ( research* W/2 ( diffus* OR disseminat* OR exchang* OR transfer* OR translation* OR application OR implement* OR mobil* OR transfer* OR uptak* OR utili* ) ) ) ) ) AND ( TITLE-ABS-KEY ( ( mental* OR well-being OR wellbeing OR psycho* OR psychiatr* OR resilience OR behaviour* OR behavior* OR stress* OR anxiety OR anxious OR panic* OR phobi* OR mood* OR depress* OR "adjustment disorder*" OR trauma* OR ( emotional W/2 ( adjustment* OR disorder* ) ) ) ) ) AND ( TITLE-ABS-KEY ( school* OR "social services" OR communit* OR advocacy* OR commission* OR policy OR policies OR "local authorit*" OR "public health" OR justice OR welfare OR educat* OR local OR district OR college OR charity OR "charitable organi*" OR "classroom-based" OR "whole-school" OR "voluntary sector*" OR "voluntary organi*" OR "third sector" OR third-sector OR non-profit* OR "not for profit" OR "social enterprise*" OR tso* OR cbo* OR vco* OR vcs* OR "civic sector*" OR "civil sector*" OR "social sector*" OR cso* OR "non-government*" OR ngo* OR npo* OR "civil society" OR ccg* ) ) AND ( TITLE-ABS-KEY ( barrier* OR facilitator* OR ( program* W/2 ( evaluat* OR acceptability OR appropriateness OR effectiveness OR efficacy OR feasibility OR impact OR sustainability ) ) OR ( implement* OR adopt* ) ) ) AND ( ( TITLE-ABS-KEY ( "national health service*" OR nhs* ) ) OR ( TITLE-ABS-KEY ( english AND NOT ( ( published OR publication* OR translat* OR written OR language* OR speak* OR literature OR citation* ) W/5 english ) ) ) OR ( TITLE-ABS-KEY ( ( gb OR "g.b." OR britain* OR ( british* AND not "british columbia" ) OR uk OR "u.k." OR united AND kingdom* OR ( england* AND not "new england" ) OR northern AND ireland* OR northern AND irish* OR scotland* OR scottish* OR ( ( wales OR "south wales" ) not "new south wales" ) OR welsh* ) ) ) OR ( ( TITLE-ABS-KEY ( ( bath OR "bath's" OR ( ( birmingham AND NOT alabama* ) OR ( "birmingham's" AND NOT alabama* ) OR bradford OR "bradford's" OR brighton OR "brighton's" OR bristol OR "bristol's" OR carlisle* OR "carlisle's" OR ( cambridge AND NOT ( massachusetts* OR boston* OR harvard* ) ) OR ( "cambridge's" AND NOT ( massachusetts* OR boston* OR harvard* ) ) OR ( canterbury AND NOT zealand* ) OR ( "canterbury's" AND NOT zealand* ) OR chelmsford OR "chelmsford's" OR chester OR "chester's" OR chichester OR "chichester's" OR coventry OR "coventry's" OR derby OR "derby's" OR ( durham AND NOT ( carolina* OR nc ) ) OR ( "durham's" AND NOT ( carolina* OR nc ) ) OR ely OR "ely's" OR exeter OR "exeter's" OR gloucester OR "gloucester's" OR hereford OR "hereford's" OR hull OR "hull's" OR lancaster OR "lancaster's" OR leeds* OR leicester OR "leicester's" OR ( lincoln AND NOT nebraska* ) OR ( "lincoln's" AND NOT nebraska* ) OR ( liverpool AND NOT ( new AND south AND wales* OR nsw ) ) OR ( "liverpool's" AND NOT ( new AND south AND wales* OR nsw ) ) OR ( ( london AND NOT ( ontario* OR ont OR toronto* ) ) OR ( "london's" AND NOT ( ontario* OR ont OR toronto* ) ) OR manchester OR "manchester's" OR ( newcastle AND NOT ( new AND south AND wales* OR nsw ) ) OR ( "newcastle's" AND NOT ( new AND south AND wales* OR nsw ) ) OR norwich OR "norwich's" OR nottingham OR "nottingham's" OR oxford OR "oxford's" OR peterborough OR "peterborough's" OR plymouth OR "plymouth's" OR portsmouth OR "portsmouth's" OR preston OR "preston's" OR ripon OR "ripon's" OR salford OR "salford's" OR salisbury OR "salisbury's" OR sheffield OR "sheffield's" OR southampton OR "southampton's" OR st AND albans OR stoke OR "stoke's" OR sunderland OR "sunderland's" OR truro OR "truro's" OR wakefield OR "wakefield's" OR wells OR westminster OR "westminster's" OR winchester OR "winchester's" OR wolverhampton OR "wolverhampton's" OR ( worcester AND NOT ( massachusetts* OR boston* OR harvard* ) ) OR ( "worcester's" AND NOT ( massachusetts* OR boston* OR harvard* ) ) OR ( york AND NOT ( "new york*" OR ny OR ontario* OR ont OR toronto* ) ) OR ( "york's" AND NOT ( "new york*" OR ny OR ontario* OR ont OR toronto* ) ) ) ) ) ) OR TITLE-ABS-KEY ( bangor OR "bangor's" OR cardiff OR "cardiff's" OR newport OR "newport's" OR st AND asaph OR "st asaph's" OR st AND davids OR swansea OR "swansea's" ) OR TITLE-ABS-KEY ( aberdeen OR "aberdeen's" OR dundee OR "dundee's" OR edinburgh OR "edinburgh's" OR glasgow OR "glasgow's" OR inverness OR ( perth AND NOT australia* ) OR ( "perth's" AND NOT australia* ) OR stirling OR "stirling's" ) OR TITLE-ABS-KEY ( armagh OR "armagh's" OR belfast OR "belfast's" OR lisburn OR "lisburn's" OR londonderry OR "londonderry's" OR derry OR "derry's" OR newry OR "newry's" ) ) ) ) AND ( LIMIT-TO ( PUBYEAR , 2021 ) OR LIMIT-TO ( PUBYEAR , 2020 ) OR LIMIT-TO ( PUBYEAR , 2019 ) OR LIMIT-TO ( PUBYEAR , 2018 ) OR LIMIT-TO ( PUBYEAR , 2017 ) OR LIMIT-TO ( PUBYEAR , 2016 ) OR LIMIT-TO ( PUBYEAR , 2015 ) OR LIMIT-TO ( PUBYEAR , 2014 ) OR LIMIT-TO ( PUBYEAR , 2013 ) OR LIMIT-TO ( PUBYEAR , 2012 ) OR LIMIT-TO ( PUBYEAR , 2011 ) ) AND ( LIMIT-TO ( LANGUAGE , "English" ) )

**Web of Science All Databases**

#1 infan* or newborn* or new-born* or perinat* or neonat* or baby* or babies or toddler* or minor* or boy* or girl* or kid or kids or child* or adolescen* or juvenil* or youth* or teen* or pubescen* or pediatric* or paediatric* or peadiatric* or school* or prematur* or preterm* or "pre term*" or prepub* or pre-pub* or preschool* or pre-school* or kindergarten* or nursery* or preadolescen* or pre-adolescen* or "young people" or "young person*" or "young adult*" or "young man" or "young men" or "young woman" or "young women" (Topic)

#2 ((research or evidence) near/2 (action or practice)) or (knowledge near/2 (application or broke* or creation or diffus* or disseminat* or exchang* or implement* or management or mobili* or translat* or transfer* or uptak* or utili*)) or (evidence* near/2 (exchang* or translat* or transfer* or diffus* or disseminat* or exchang* or implement* or management or mobil* or uptak* or utili*)) or (research* near/2 (diffus* or disseminat* or exchang* or transfer* or translation* or application or implement* or mobil* or transfer* or uptak* or utili*)) (Topic)

#3 mental* or well-being or wellbeing or psycho* or psychiatr* or resilience or behaviour* or behavior* or stress* or anxiety or anxious or panic* or phobi* or mood* or depress* or "adjustment disorder*" or trauma* or (emotional near/2 (adjustment* or disorder*)) (Topic)

#4 school* or "social services" or communit* or advocacy* or commission* or policy or policies or "local authorit*" or "public health" or justice or welfare or educat* or local or district or college or charity or "charitable organi*" or "classroom-based" or "whole-school" or "voluntary sector*" or "voluntary organi*" or "third sector" or third-sector or non-profit* or "not for profit" or "social enterprise*" or TSO* or CBO* or VCO* or VCS* or "civic sector*" or "civil sector*" or "social sector*" or CSO* or "non-government*" or NGO* or NPO* or "civil society" or CCG* (Topic)

#5 barrier* or facilitator* or (program* near/2 (evaluat* or acceptability or appropriateness or effectiveness or efficacy or feasibility or impact or sustainability)) or implement* or adopt* (Topic)

#6 "national health service*" or nhs* (Topic) or (english not ((published or publication* or translat* or written or language* or speak* or literature or citation*) near/5 english)) (Topic) or (gb or "g.b." or britain* or (british* not "british columbia") or uk or "u.k." or united kingdom* or (england* not "new england") or northern ireland* or northern irish* or scotland* or scottish* or ((wales or "south wales") not "new south wales") or welsh*) (Topic) or (bath or "bath's" or ((birmingham not alabama*) or ("birmingham's" not alabama*) or bradford or "bradford's" or brighton or "brighton's" or bristol or "bristol's" or carlisle* or "carlisle's" or (cambridge not (massachusetts* or boston* or harvard*)) or ("cambridge's" not (massachusetts* or boston* or harvard*)) or (canterbury not zealand*) or ("canterbury's" not zealand*) or chelmsford or "chelmsford's" or chester or "chester's" or chichester or "chichester's" or coventry or "coventry's" or derby or "derby's" or (durham not (carolina* or nc)) or ("durham's" not (carolina* or nc)) or ely or "ely's" or exeter or "exeter's" or gloucester or "gloucester's" or hereford or "hereford's" or hull or "hull's" or lancaster or "lancaster's" or leeds* or leicester or "leicester's" or (lincoln not nebraska*) or ("lincoln's" not nebraska*) or (liverpool not (new south wales* or nsw)) or ("liverpool's" not (new south wales* or nsw)) or ((london not (ontario* or ont or toronto*)) or ("london's" not (ontario* or ont or toronto*)) or manchester or "manchester's" or (newcastle not (new south wales* or nsw)) or ("newcastle's" not (new south wales* or nsw)) or norwich or "norwich's" or nottingham or "nottingham's" or oxford or "oxford's" or peterborough or "peterborough's" or plymouth or "plymouth's" or portsmouth or "portsmouth's" or preston or "preston's" or ripon or "ripon's" or salford or "salford's" or salisbury or "salisbury's" or sheffield or "sheffield's" or southampton or "southampton's" or st albans or stoke or "stoke's" or sunderland or "sunderland's" or truro or "truro's" or wakefield or "wakefield's" or wells or westminster or "westminster's" or winchester or "winchester's" or wolverhampton or "wolverhampton's" or (worcester not (massachusetts* or boston* or harvard*)) or ("worcester's" not (massachusetts* or boston* or harvard*)) or (york not ("new york*" or ny or ontario* or ont or toronto*)) or ("york's" not ("new york*" or ny or ontario* or ont or toronto*))))) (Topic) or (bangor or "bangor's" or cardiff or "cardiff's" or newport or "newport's" or st asaph or "st asaph's" or st davids or swansea or "swansea's") (Topic) or (aberdeen or "aberdeen's" or dundee or "dundee's" or edinburgh or "edinburgh's" or glasgow or "glasgow's" or inverness or (perth not australia*) or ("perth's" not australia*) or stirling or "stirling's") (Topic) or (armagh or "armagh's" or belfast or "belfast's" or lisburn or "lisburn's" or londonderry or "londonderry's" or derry or "derry's" or newry or "newry's") (Topic)

#7 #1 and #2 and #3 and #4 and #5 and 2021 or 2020 or 2019 or 2018 or 2017 or 2016 or 2015 or 2014 or 2013 or 2012 or 2011 (Publication Years)

#8 #1 and #2 and #3 and #4 and #5 and 2021 or 2020 or 2019 or 2018 or 2017 or 2016 or 2015 or 2014 or 2013 or 2012 or 2011 (Publication Years) and English (Languages)

**EBSCO CINAHL**

| **#** | **Query** | **Results** |
| --- | --- | --- |
| S16 | S1 AND S2 AND S3 AND S4 AND S5 AND S13 | 426 |
| S15 | S1 AND S2 AND S3 AND S4 AND S5 AND S13 | 427 |
| S14 | S1 AND S2 AND S3 AND S4 AND S5 AND S13 | 507 |
| S13 | S6 OR S7 OR S8 OR S9 OR S10 OR S11 OR S12 | 745,530 |
| S12 | TI ( armagh or "armagh's" or belfast or "belfast's" or lisburn or "lisburn's" or londonderry or "londonderry's" or derry or "derry's" or newry or "newry's" ) OR AB ( armagh or "armagh's" or belfast or "belfast's" or lisburn or "lisburn's" or londonderry or "londonderry's" or derry or "derry's" or newry or "newry's" ) | 791 |
| S11 | TI ( aberdeen or "aberdeen's" or dundee or "dundee's" or edinburgh or "edinburgh's" or glasgow or "glasgow's" or inverness or (perth not australia*) or ("perth's" not australia*) or stirling or "stirling's" ) OR AB ( aberdeen or "aberdeen's" or dundee or "dundee's" or edinburgh or "edinburgh's" or glasgow or "glasgow's" or inverness or (perth not australia*) or ("perth's" not australia*) or stirling or "stirling's" ) | 13,217 |
| S10 | TI ( bangor or "bangor's" or cardiff or "cardiff's" or newport or "newport's" or st asaph or "st asaph's" or st davids or swansea or "swansea's" ) OR AB ( bangor or "bangor's" or cardiff or "cardiff's" or newport or "newport's" or st asaph or "st asaph's" or st davids or swansea or "swansea's" ) | 1,504 |
| S9 | TI ( bath or "bath's" or ((birmingham not alabama*) or ("birmingham's" not alabama*) or bradford or "bradford's" or brighton or "brighton's" or bristol or "bristol's" or carlisle* or "carlisle's" or (cambridge not (massachusetts* or boston* or harvard*)) or ("cambridge's" not (massachusetts* or boston* or harvard*)) or (canterbury not zealand*) or ("canterbury's" not zealand*) or chelmsford or "chelmsford's" or chester or "chester's" or chichester or "chichester's" or coventry or "coventry's" or derby or "derby's" or (durham not (carolina* or nc)) or ("durham's" not (carolina* or nc)) or ely or "ely's" or exeter or "exeter's" or gloucester or "gloucester's" or hereford or "hereford's" or hull or "hull's" or lancaster or "lancaster's" or leeds* or leicester or "leicester's" or (lincoln not nebraska*) or ("lincoln's" not nebraska*) or (liverpool not (new south wales* or nsw)) or ("liverpool's" not (new south wales* or nsw)) or ((london not (ontario* or ont or toronto*)) or ("london's" not (ontario* or ont or toronto*)) or manchester or "manchester's" or (newcastle not (new south wales* or nsw)) or ("newcastle's" not (new south wales* or nsw)) or norwich or "norwich's" or nottingham or "nottingham's" or oxford or "oxford's" or peterborough or "peterborough's" or plymouth or "plymouth's" or portsmouth or "portsmouth's" or preston or "preston's" or ripon or "ripon's" or salford or "salford's" or salisbury or "salisbury's" or sheffield or "sheffield's" or southampton or "southampton's" or st albans or stoke or "stoke's" or sunderland or "sunderland's" or truro or "truro's" or wakefield or "wakefield's" or wells or westminster or "westminster's" or winchester or "winchester's" or wolverhampton or "wolverhampton's" or (worcester not (massachusetts* or boston* or harvard*)) or ("worcester's" not (massachusetts* or boston* or harvard*)) or (york not ("new york*" or ny or ontario* or ont or toronto*)) or ("york's" not ("new york*" or ny or ontario* or ont or toronto*)))) ) OR AB ( bath or "bath's" or ((birmingham not alabama*) or ("birmingham's" not alabama*) or bradford or "bradford's" or brighton or "brighton's" or bristol or "bristol's" or carlisle* or "carlisle's" or (cambridge not (massachusetts* or boston* or harvard*)) or ("cambridge's" not (massachusetts* or boston* or harvard*)) or (canterbury not zealand*) or ("canterbury's" not zealand*) or chelmsford or "chelmsford's" or chester or "chester's" or chichester or "chichester's" or coventry or "coventry's" or derby or "derby's" or (durham not (carolina* or nc)) or ("durham's" not (carolina* or nc)) or ely or "ely's" or exeter or "exeter's" or gloucester or "gloucester's" or hereford or "hereford's" or hull or "hull's" or lancaster or "lancaster's" or leeds* or leicester or "leicester's" or (lincoln not nebraska*) or ("lincoln's" not nebraska*) or (liverpool not (new south wales* or nsw)) or ("liverpool's" not (new south wales* or nsw)) or ((london not (ontario* or ont or toronto*)) or ("london's" not (ontario* or ont or toronto*)) or manchester or "manchester's" or (newcastle not (new south wales* or nsw)) or ("newcastle's" not (new south wales* or nsw)) or norwich or "norwich's" or nottingham or "nottingham's" or oxford or "oxford's" or peterborough or "peterborough's" or plymouth or "plymouth's" or portsmouth or "portsmouth's" or preston or "preston's" or ripon or "ripon's" or salford or "salford's" or salisbury or "salisbury's" or sheffield or "sheffield's" or southampton or "southampton's" or st albans or stoke or "stoke's" or sunderland or "sunderland's" or truro or "truro's" or wakefield or "wakefield's" or wells or westminster or "westminster's" or winchester or "winchester's" or wolverhampton or "wolverhampton's" or (worcester not (massachusetts* or boston* or harvard*)) or ("worcester's" not (massachusetts* or boston* or harvard*)) or (york not ("new york*" or ny or ontario* or ont or toronto*)) or ("york's" not ("new york*" or ny or ontario* or ont or toronto*)))) ) | 571,732 |
| S8 | TI ( gb or "g.b." or britain* or (british* not "british columbia") or uk or "u.k." or united kingdom* or (england* not "new england") or northern ireland* or northern irish* or scotland* or scottish* or ((wales or "south wales") not "new south wales") or welsh* ) OR AB ( gb or "g.b." or britain* or (british* not "british columbia") or uk or "u.k." or united kingdom* or (england* not "new england") or northern ireland* or northern irish* or scotland* or scottish* or ((wales or "south wales") not "new south wales") or welsh* ) | 159,698 |
| S7 | TI ( english not ((published or publication* or translat* or written or language* or speak* or literature or citation*) N5 english) ) OR AB ( english not ((published or publication* or translat* or written or language* or speak* or literature or citation*) N5 english) ) | 17,393 |
| S6 | TI ( "national health service*" or nhs* ) OR AB ( "national health service*" or nhs* ) | 42,483 |
| S5 | TI ( barrier* or facilitator* or (program* N2 (evaluat* or acceptability or appropriateness or effectiveness or efficacy or feasibility or impact or sustainability)) or implement* or adopt* ) OR AB ( barrier* or facilitator* or (program* N2 (evaluat* or acceptability or appropriateness or effectiveness or efficacy or feasibility or impact or sustainability)) or implement* or adopt* ) | 364,048 |
| S4 | TI ( school* or "social services" or communit* or advocacy* or commission* or policy or policies or "local authorit*" or "public health" or justice or welfare or educat* or local or district or college or charity or "charitable organi*" or "classroom-based" or "whole-school" or "voluntary sector*" or "voluntary organi*" or "third sector" or third-sector or non-profit* or "not for profit" or "social enterprise*" or TSO* or CBO* or VCO* or VCS* or "civic sector*" or "civil sector*" or "social sector*" or CSO* or "non-government*" or NGO* or NPO* or "civil society" or CCG* ) OR AB ( school* or "social services" or communit* or advocacy* or commission* or policy or policies or "local authorit*" or "public health" or justice or welfare or educat* or local or district or college or charity or "charitable organi*" or "classroom-based" or "whole-school" or "voluntary sector*" or "voluntary organi*" or "third sector" or third-sector or non-profit* or "not for profit" or "social enterprise*" or TSO* or CBO* or VCO* or VCS* or "civic sector*" or "civil sector*" or "social sector*" or CSO* or "non-government*" or NGO* or NPO* or "civil society" or CCG* ) | 1,060,641 |
| S3 | TI ( mental* or well-being or wellbeing or psycho* or psychiatr* or resilience or behaviour* or behavior* or stress* or anxiety or anxious or panic* or phobi* or mood* or depress* or "adjustment disorder*" or trauma* or (emotional N2 (adjustment* or disorder*)) ) OR AB ( mental* or well-being or wellbeing or psycho* or psychiatr* or resilience or behaviour* or behavior* or stress* or anxiety or anxious or panic* or phobi* or mood* or depress* or "adjustment disorder*" or trauma* or (emotional N2 (adjustment* or disorder*)) ) | 1,032,457 |
| S2 | TI ( ((research or evidence) N2 (action or practice)) or (knowledge N2 (application or broke* or creation or diffus* or disseminat* or exchang* or implement* or management or mobili* or translat* or transfer* or uptak* or utili*)) or (evidence* N2 (exchang* or translat* or transfer* or diffus* or disseminat* or exchang* or implement* or management or mobil* or uptak* or utili*)) or (research* N2 (diffus* or disseminat* or exchang* or transfer* or translation* or application or implement* or mobil* or transfer* or uptak* or utili*)) ) OR AB ( ((research or evidence) N2 (action or practice)) or (knowledge N2 (application or broke* or creation or diffus* or disseminat* or exchang* or implement* or management or mobili* or translat* or transfer* or uptak* or utili*)) or (evidence* N2 (exchang* or translat* or transfer* or diffus* or disseminat* or exchang* or implement* or management or mobil* or uptak* or utili*)) or (research* N2 (diffus* or disseminat* or exchang* or transfer* or translation* or application or implement* or mobil* or transfer* or uptak* or utili*)) ) | 94,715 |
| S1 | TI ( infan* or newborn* or new-born* or perinat* or neonat* or baby* or babies or toddler* or minor* or boy* or girl* or kid or kids or child* or adolescen* or juvenil* or youth* or teen* or pubescen* or pediatric* or paediatric* or peadiatric* or school* or prematur* or preterm* or "pre term*" or prepub* or pre-pub* or preschool* or pre-school* or kindergarten* or nursery* or preadolescen* or pre-adolescen* or "young people" or "young person*" or "young adult*" or "young man" or "young men" or "young woman" or "young women" ) OR AB ( infan* or newborn* or new-born* or perinat* or neonat* or baby* or babies or toddler* or minor* or boy* or girl* or kid or kids or child* or adolescen* or juvenil* or youth* or teen* or pubescen* or pediatric* or paediatric* or peadiatric* or school* or prematur* or preterm* or "pre term*" or prepub* or pre-pub* or preschool* or pre-school* or kindergarten* or nursery* or preadolescen* or pre-adolescen* or "young people" or "young person*" or "young adult*" or "young man" or "young men" or "young woman" or "young women" ) | 1,072,054 |

**EBSCO ERIC**

| **#** | **Query** | **Results** |
| --- | --- | --- |
| S16 | S1 AND S2 AND S3 AND S4 AND S5 AND S13 | 2,505 |
| S15 | S1 AND S2 AND S3 AND S4 AND S5 AND S13 | 2,507 |
| S14 | S1 AND S2 AND S3 AND S4 AND S5 AND S13 | 3,902 |
| S13 | S6 OR S7 OR S8 OR S9 OR S10 OR S11 OR S12 | 1,486,153 |
| S12 | TX armagh or "armagh's" or belfast or "belfast's" or lisburn or "lisburn's" or londonderry or "londonderry's" or derry or "derry's" or newry or "newry's" | 369 |
| S11 | TX aberdeen or "aberdeen's" or dundee or "dundee's" or edinburgh or "edinburgh's" or glasgow or "glasgow's" or inverness or (perth not australia*) or ("perth's" not australia*) or stirling or "stirling's" | 3,694 |
| S10 | TX bangor or "bangor's" or cardiff or "cardiff's" or newport or "newport's" or st asaph or "st asaph's" or st davids or swansea or "swansea's" | 844 |
| S9 | TX bath or "bath's" or ((birmingham not alabama*) or ("birmingham's" not alabama*) or bradford or "bradford's" or brighton or "brighton's" or bristol or "bristol's" or carlisle* or "carlisle's" or (cambridge not (massachusetts* or boston* or harvard*)) or ("cambridge's" not (massachusetts* or boston* or harvard*)) or (canterbury not zealand*) or ("canterbury's" not zealand*) or chelmsford or "chelmsford's" or chester or "chester's" or chichester or "chichester's" or coventry or "coventry's" or derby or "derby's" or (durham not (carolina* or nc)) or ("durham's" not (carolina* or nc)) or ely or "ely's" or exeter or "exeter's" or gloucester or "gloucester's" or hereford or "hereford's" or hull or "hull's" or lancaster or "lancaster's" or leeds* or leicester or "leicester's" or (lincoln not nebraska*) or ("lincoln's" not nebraska*) or (liverpool not (new south wales* or nsw)) or ("liverpool's" not (new south wales* or nsw)) or ((london not (ontario* or ont or toronto*)) or ("london's" not (ontario* or ont or toronto*)) or manchester or "manchester's" or (newcastle not (new south wales* or nsw)) or ("newcastle's" not (new south wales* or nsw)) or norwich or "norwich's" or nottingham or "nottingham's" or oxford or "oxford's" or peterborough or "peterborough's" or plymouth or "plymouth's" or portsmouth or "portsmouth's" or preston or "preston's" or ripon or "ripon's" or salford or "salford's" or salisbury or "salisbury's" or sheffield or "sheffield's" or southampton or "southampton's" or st albans or stoke or "stoke's" or sunderland or "sunderland's" or truro or "truro's" or wakefield or "wakefield's" or wells or westminster or "westminster's" or winchester or "winchester's" or wolverhampton or "wolverhampton's" or (worcester not (massachusetts* or boston* or harvard*)) or ("worcester's" not (massachusetts* or boston* or harvard*)) or (york not ("new york*" or ny or ontario* or ont or toronto*)) or ("york's" not ("new york*" or ny or ontario* or ont or toronto*)))) | 237,668 |
| S8 | TX gb or "g.b." or britain* or (british* not "british columbia") or uk or "u.k." or united kingdom* or (england* not "new england") or northern ireland* or northern irish* or scotland* or scottish* or ((wales or "south wales") not "new south wales") or welsh* | 98,508 |
| S7 | TX english not ((published or publication* or translat* or written or language* or speak* or literature or citation*) N5 english) | 1,439,525 |
| S6 | TX "national health service*" or nhs* | 678 |
| S5 | TX barrier* or facilitator* or (program* N2 (evaluat* or acceptability or appropriateness or effectiveness or efficacy or feasibility or impact or sustainability)) or implement* or adopt* | 316,757 |
| S4 | TX school* or "social services" or communit* or advocacy* or commission* or policy or policies or "local authorit*" or "public health" or justice or welfare or educat* or local or district or college or charity or "charitable organi*" or "classroom-based" or "whole-school" or "voluntary sector*" or "voluntary organi*" or "third sector" or third-sector or non-profit* or "not for profit" or "social enterprise*" or TSO* or CBO* or VCO* or VCS* or "civic sector*" or "civil sector*" or "social sector*" or CSO* or "non-government*" or NGO* or NPO* or "civil society" or CCG* | 1,603,812 |
| S3 | TX mental* or well-being or wellbeing or psycho* or psychiatr* or resilience or behaviour* or behavior* or stress* or anxiety or anxious or panic* or phobi* or mood* or depress* or "adjustment disorder*" or trauma* or (emotional N2 (adjustment* or disorder*)) | 476,519 |
| S2 | TX ((research or evidence) N2 (action or practice)) or (knowledge N2 (application or broke* or creation or diffus* or disseminat* or exchang* or implement* or management or mobili* or translat* or transfer* or uptak* or utili*)) or (evidence* N2 (exchang* or translat* or transfer* or diffus* or disseminat* or exchang* or implement* or management or mobil* or uptak* or utili*)) or (research* N2 (diffus* or disseminat* or exchang* or transfer* or translation* or application or implement* or mobil* or transfer* or uptak* or utili*)) | 70,497 |
| S1 | TX infan* or newborn* or new-born* or perinat* or neonat* or baby* or babies or toddler* or minor* or boy* or girl* or kid or kids or child* or adolescen* or juvenil* or youth* or teen* or pubescen* or pediatric* or paediatric* or peadiatric* or school* or prematur* or preterm* or "pre term*" or prepub* or pre-pub* or preschool* or pre-school* or kindergarten* or nursery* or preadolescen* or pre-adolescen* or "young people" or "young person*" or "young adult*" or "young man" or "young men" or "young woman" or "young women" | 1,006,341 |
